# Heat Shock Proteins as Targets for Cancer Therapeutics

**DOI:** 10.3390/jcm15103605

**Published:** 2026-05-08

**Authors:** Aryaman Trikala, Binghui Shen, Sharonlin Bhardwaj

**Affiliations:** 1 School of Medicine, California University of Science and Medicine, Colton, CA 92324, USA; aryaman.trikala@md.cusm.edu; 2 Department of Cancer Genetics & Epigenetics, Beckman Research Institute, City of Hope, 1500 East Duarte Road, Duarte, CA 91010, USA; bshen@coh.org; 3 Department of Medical Oncology, City of Hope Medical Center, Duarte, CA 91010, USA

**Keywords:** heat shock protein, cancer, therapeutics

## Abstract

**Introduction**: Heat shock proteins (HSPs) are stress-responsive molecular chaperones that are frequently dysregulated in cancer and contribute to tumorigenesis, invasion, metastasis, immune interactions, and resistance to therapy. Distinct HSP families, including HSP27, HSP60, HSP70, HSP90, and HSP110, promote malignant progression through complementary effects on apoptosis regulation, mitochondrial function, proteostasis, and stabilization of oncogenic signaling pathways. This makes HSPs attractive therapeutic targets. Their coordinated roles within stress-adaptive chaperone networks further garner interest in targeting multiple HSP families in cancer therapy. **Discussion**: Preclinical and clinical studies have established multiple HSP families as promising anticancer targets; however, clinical translation of HSP-directed therapies has been challenged by toxicity, compensatory heat shock responses, and resistance mechanisms. Many N-terminal HSP90 inhibitors have shown clinical utility but have also highlighted the need for alternative approaches, including C-terminal inhibition, HSP70-directed therapies, and rational combination strategies targeting compensatory survival pathways. Emerging inhibitors targeting HSP27, HSP60, and HSP110, as well as HSP-based vaccines, further expand therapeutic opportunities across cancer subtypes. Collectively, these approaches highlight the growing therapeutic relevance of disrupting interconnected HSP networks rather than targeting individual chaperones in isolation. **Conclusions**: Future development of heat shock protein-targeted therapies will require a deeper understanding of HSP-mediated chemoresistance. Clinical trial and drug development approaches may benefit from combination or multi-targeted strategies that simultaneously disrupt multiple components of the heat shock protein network to achieve more durable anticancer responses.

## 1. Introduction

Heat shock proteins (HSPs) are a class of ubiquitous chaperone proteins that are upregulated in cellular states of stress. They typically show cryoprotective properties by preserving cellular processes, preventing misfolding and denaturation, and promoting renaturation of other proteins. In times of extreme cellular stress, a natural response includes activation of proapoptotic signaling pathways. HSPs also prevent activation of proapoptotic signals [1]. The structure of these molecular chaperones is highly conserved throughout evolution and across species. They are classified into five families based on their molecular weight, expressed in kilodaltons (kDa): HSP27, HSP60, HSP70, HSP90, and HSP110 [2]. All HSPs play a role in signal transduction for growth and development; however, under stress or disease, HSP levels may vary, leading to dysregulation of proliferation and cell death [2].

**Figure 1:**
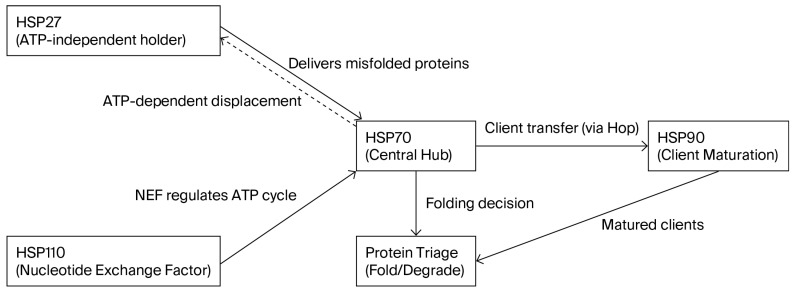
Schematic illustrating the coordinated roles of HSP27, HSP70, HSP90, and HSP110 in protein quality control. The dashed arrow represents ATP-dependent client transfer from HSP27 to HSP70, distinguishing this auxiliary regulatory interaction from the primary substrate maturation pathway.

Specifically, in tumor development, HSPs play a critical role by supporting various hallmarks of cancer. Under healthy conditions, HSPs maintain protein homeostasis through their role in folding, assembly, and degradation of misfolded proteins [3]. However, in malignant conditions, the same HSPs are co-opted to contribute to the stabilization, folding, and functional regulation of oncogenic proteins. This effectively promotes further tumor growth and survival by allowing them to avoid programmed cell death [4]. Table 1 details the specific cancer-related roles of the different HSP families based on their physiological function. Figure 1 illustrates the functional relationships among HSP families, highlighting coordination within the chaperone network.

This narrative review focuses on key preclinical and clinical studies evaluating heat shock protein inhibition across major cancer types, with an emphasis on representative mechanisms of action and therapeutic development. Studies were selected based on their relevance to clinical translation, mechanistic insights, and citation frequency within the field.

## 2. Heat Shock Protein Families in Human Diseases

### 2.1. HSP90

HSP90 is a ubiquitous ATP-dependent molecular chaperone composed of subunits containing N-terminal, middle, and C-terminal domains that regulate ATP binding, co-chaperone interactions, and dimerization. HSP90 plays an indispensable role in the late folding and maturation of signaling proteins, including steroid hormone receptors, kinases, oncoproteins, and tumor suppressors [14]. By stabilizing these client proteins, HSP90 contributes to proteostasis and to the regulation of signaling pathways involved in proliferation and survival.

In cancer, HSP90 is frequently overexpressed, and elevated expression has been associated with tumor progression, aggressive disease biology, and poor clinical outcomes. In breast cancer, increased HSP90 expression has been correlated with more malignant disease, reduced treatment responsiveness, and worse overall survival [15]. In addition, HSP90 may function as a biochemical buffer for mutated oncoproteins, stabilizing and maintaining their function, thereby contributing to malignant progression and resistance to apoptosis [16].

### 2.2. HSP70

HSP70 is an ATP-dependent molecular chaperone composed of an ATPase domain and a substrate-binding domain connected by a flexible linker that enables allosteric signaling. Through coordinated interactions with co-chaperones, including J-domain proteins (HSP40) and nucleotide exchange factors, HSP70 regulates protein folding, prevents aggregation, and supports recovery from cellular stress [14]. Through these functions, HSP70 contributes to proteostasis, stress adaptation, and maintenance of cellular homeostasis.

In malignant cells, HSP70 overexpression promotes survival by suppressing apoptosis and enhancing tolerance to proteotoxic stress. Elevated HSP70 expression has been reported across numerous malignancies, including lung, colorectal, breast, pancreatic, glioblastoma, sarcomas, and hematologic cancers. In pancreatic cancer, HSP70 expression has been reported to be markedly increased relative to normal tissue, and experimental downregulation of HSP70 has been shown to trigger apoptotic cell death [17,18,19]. Emerging evidence has also implicated HSP70 in adaptive pathways relevant to cancer biology, including a potential role in modulating cellular susceptibility to cuproptosis, a copper-dependent form of regulated cell death [20].

### 2.3. HSP110

HSP110 is a nonclassical member of the HSP70-associated family that functions as a major nucleotide exchange factor (NEF) for HSP70 [21]. HSP110 catalyzes the release of ADP from HSP70’s nucleotide-binding domain, enabling ATP binding and completing the HSP70 chaperone cycle. The four isoforms of HSP110—HSPH1, HSPH2, HSPH3, and Grp180—all share structural features that distinguish them from canonical HSP70 proteins [22]. As a non-canonical HSP70 family member, HSP110 primarily functions as a holdase chaperone, whereas the canonical HSP70 proteins function as refoldases, contributing to proteostasis and adaptive stress responses [23]. These structural and functional distinctions have supported growing interest in the role of HSP110 in cancer biology.

Elevated HSP110 expression levels are associated with chemotherapy resistance and adverse prognosis [24]. Beyond these expression-based associations, HSP110 has been implicated in tumor progression through promotion of oncogenic signaling, including activation of STAT3-mediated pathways in colon cancer. Notably, inactivating mutations, such as HSP110ΔE9, in microsatellite instability tumors have been associated with improved chemotherapy response and a more favorable prognosis, further supporting a functional role for HSP110 in malignant biology [25].

### 2.4. HSP27

HSP27 (HSPB1), a small heat shock protein (sHSP), functions as an ATP-independent chaperone that delivers misfolded protein substrates to HSP70 [26]. This interaction occurs through a sequential mechanism: HSP27 initially binds to early-unfolding protein intermediates and sequesters them in large HSP27-substrate complexes, preventing uncontrolled aggregation [21,27]. HSP70 then actively displaces HSP27 from these assemblies, binds to the substrate core, and directs proteins toward refolding pathways. Through these functions, HSP27 contributes to cytoskeletal stability and protection against stress-induced apoptosis.

HSP27 dysregulation and overexpression have been reported across multiple malignancies, including breast, prostate, gastric, ovarian, lung, colorectal, and pancreatic cancers [28]. In malignant cells, HSP27 has been implicated in tumor progression through suppression of apoptosis, promotion of treatment resistance, and regulation of cytoskeletal reorganization associated with invasion and metastatic behavior. Through these functions, HSP27 may support cancer cell survival and contribute to malignant progression, underscoring its relevance in tumor biology.

### 2.5. HSP60

HSP60 is a mitochondrial chaperonin that functions together with its co-chaperonin HSP10 to facilitate the folding of newly imported mitochondrial proteins and maintain mitochondrial proteostasis. Although classically localized in the mitochondria, HSP60 has also been identified in the cytosol, nucleus, and extracellular space, where it may exert additional chaperone-dependent and independent functions. Beyond its role in protein homeostasis, mutations, dysregulated expression, and altered subcellular localization of HSP60 have been implicated in diverse disease states, including neurodegenerative, inflammatory, and malignant diseases [29].

HSP60 overexpression has been implicated in tumor progression through support of mitochondrial function and maintenance of pro-survival signaling pathways. In pancreatic ductal adenocarcinoma, elevated HSP60 expression has been associated with tumor growth through ATP-dependent ERK1/2 signaling, while experimental knockdown of HSP60 has been shown to induce apoptosis and cell cycle arrest [30]. These findings support a functional role for HSP60 in malignant biology and therapeutic resistance.

Collectively, the diverse roles of heat shock protein families in stress adaptation, proteostasis, and disease progression have made them attractive therapeutic targets. These biological functions have motivated the development of multiple pharmacologic strategies to disrupt heat shock protein-mediated mechanisms of disease progression, as discussed in the following sections.

## 3. Heat Shock Protein Inhibitors

In this section, representative inhibitors are discussed according to their mechanism of action, developmental stage, tumor relevance, and clinical limitations to facilitate comparison across compounds and protein families.

### 3.1. HSP90 Inhibitors

Given the central role of HSP90 in maintaining oncogenic proteostasis, multiple pharmacologic strategies have been developed to inhibit its chaperone function. HSP90 inhibitors can be widely categorized into first-generation natural products, second-generation synthetic derivatives, and emerging isoform-selective or C-terminal inhibitors, each with distinct pharmacologic and clinical profiles.

First-generation HSP90 inhibitors were primarily derived from naturally occurring compounds, including geldanamycin and radicicol. Geldanamycin, an antibiotic originally discovered in *Streptomyces hygroscopicus*, serves as a competitive inhibitor of HSP90 at the N-terminal binding pocket. Although preclinical studies demonstrated effective HSP90 inhibition, its clinical development was limited by high levels of hepatotoxicity and inadequate solubility, preventing its advancement into clinical use. To address these limitations, several derivatives have been developed, including 17-allylamino-17-demethoxygeldanamycin (17-AAG), which demonstrated improved pharmacologic properties and was evaluated in early clinical trials [31]. Notably, 17-AAG has been extensively studied in melanoma, where oncogenic drivers, including mutant BRAF, exhibit dependence on HSP90 for stability [32]. Early-phase trials demonstrated some therapeutic benefit, particularly in combination regimens. For example, 17-AAG was evaluated with trastuzumab in HER2-positive metastatic breast cancer because HER2 is a highly sensitive HSP90 client protein, and HSP90 inhibition promotes proteasomal degradation of HER2 and other growth factor signaling proteins. In a phase II study of patients with trastuzumab-refractory HER2-positive metastatic breast cancer, the combination demonstrated objective responses, with a clinical benefit rate of 59% [33]. Additional phase I combination studies evaluated 17-AAG with sorafenib, a Raf kinase inhibitor, based on the rationale that HSP90 inhibition affects Raf kinase signaling and may enhance sorafenib-mediated antitumor activity. This combination demonstrated clinical and pharmacodynamic activity in kidney cancer and melanoma, although dose-limiting toxicities included transaminitis and hand-foot syndrome [34]. However, poor water solubility, hepatotoxicity, and inconsistent efficacy ultimately limited further clinical development of 17-AAG [11].

Radicicol is another naturally occurring macrolide antibiotic derived from the *Monosporium bonorden* fungus. Radicicol’s mechanism is similar to that of geldanamycin, although radicicol has a much higher binding affinity for HSP90. Radicicol contributes to the degradation of NQO1, a flavoenzyme that functions to stabilize the tumor suppressor protein p53 [35]. In vitro preclinical studies were promising; however, clinical translation has been limited by low in vivo biological stability. As a result, radicicol derivatives are now being developed and studied for biological stability and clinical efficacy [15].

To overcome the limitations of first-generation HSP90 inhibitors, several second-generation and synthetic HSP90 inhibitors have been developed with improved pharmacologic properties and reduced toxicity. One of the most extensively studied second-generation HSP90 inhibitors is ganetespib (STA-9090). In contrast to earlier generations of HSP90 inhibitors, ganetespib lacks the benzoquinone moiety associated with hepatotoxicity in earlier agents [36]. Additionally, it lacks the ocular toxicity associated with many HSP90 inhibitors due to its increased rate of retinal elimination [37]. However, despite these improvements, ganetespib has been evaluated across multiple phase I–III clinical trials, and the results have shown varying levels of efficacy, significant gastrointestinal toxicity, and no improvement in survival in later-phase studies, highlighting the challenges in translating HSP90 inhibition into consistent clinical benefit [11,38].

Onalespib is a synthetic HSP90 inhibitor that has been evaluated both as a single agent and in combination regimens. In one phase I study, onalespib was combined with AT7519, a pan-CDK inhibitor, based on preclinical evidence that AT7519 may suppress compensatory HSP70 upregulation and impair pro-survival transcriptional signaling. Among 28 patients with advanced solid tumors, the regimen was tolerable and demonstrated pharmacodynamic target engagement, with partial responses observed in patients with palatal adenocarcinoma and Sertoli–Leydig cell tumors [39]. Onalespib was also evaluated in combination with erlotinib, an epidermal growth factor receptor inhibitor, in EGFR-mutant non-small cell lung cancer, based on evidence that EGFR exon 20 insertion mutations interact with and depend on HSP90 in vitro. However, the phase I/II study showed limited clinical activity, with no responses among patients with EGFR exon 20 insertion disease, and was closed early due to overlapping toxicities, predominantly diarrhea [40].

Another promising HSP90 inhibitor under study for its potential therapeutic benefits is Pimitespib (TAS-116). Unlike other HSP90 inhibitors, Pimitespib binds specifically to the cytosolic HSP90 α/β isoforms by targeting both the ATP-binding pocket and a hydrophobic sub-pocket, which makes it more selective than other HSP90 inhibitors [41]. The first-in-human phase I study, completed in February 2017, showed that Pimitespib demonstrated early signs of antitumor activity in patients with advanced solid tumors, specifically those with previously treated gastrointestinal stromal tumors (GIST) [42]. More recently, a phase III trial completed in April 2020 demonstrated a satisfactory safety profile and improved survival outcomes in patients with advanced GIST refractory to the standard tyrosine kinase inhibitors [43]. Pimitespib has been approved as a fourth-line treatment for GIST in Japan, representing one of the few HSP90 inhibitors to demonstrate meaningful clinical benefit, albeit in a limited and tumor-specific context [41].

In addition to N-terminal inhibitors, newer strategies have focused on C-terminal HSP90 inhibitors to overcome resistance mechanisms associated with earlier agents. Unlike N-terminal ATP-site inhibitors, which can trigger compensatory heat shock responses and upregulation of cytoprotective proteins such as HSP27, HSP70, HSP40, and HSP90, C-terminal HSP90 inhibitors may induce client protein degradation without activating the heat shock response. This distinction has motivated interest in C-terminal inhibition as a strategy to overcome chemoprotective resistance mechanisms [44]. NCT-58, a C-terminal HSP90 inhibitor, effectively targets trastuzumab-resistant HER2-positive breast cancer stem-like cells by downregulating HER family members and inhibiting Akt phosphorylation. NCT-58 reduces tumor growth, angiogenesis, and stem-like markers in vitro and in a xenograft model, supporting its potential for overcoming trastuzumab resistance in breast cancer [45].

Despite extensive research and development, single-agent HSP90 N-terminal inhibitors have demonstrated limited clinical utility to date. While preclinical studies highlighted their potential in targeting the chaperone function of HSP90 to disrupt multiple oncogenic signaling pathways, translating these findings into clinical success has been challenging. Issues such as off-target toxicities, poor pharmacokinetics, and the emergence of resistance mechanisms have hindered their efficacy in human trials. Importantly, compensatory upregulation of other heat shock proteins, particularly HSP70, further limits the effectiveness of HSP90 inhibition by maintaining proteostasis and promoting tumor cell survival. This highlights the functional redundancy within the heat shock protein network and underscores a key limitation of targeting HSP90 in isolation.

### 3.2. HSP70 Inhibitors

In contrast to HSP90-directed strategies, interest in HSP70 inhibition has been driven in part by adaptive resistance mechanisms, particularly compensatory upregulation of HSP70 following HSP90 blockade. This functional interplay within the heat shock protein network has positioned HSP70 as an important therapeutic target, prompting the development of several pharmacologic strategies to disrupt its pro-survival activity in cancer.

In non-small cell lung carcinoma (NSCLC), the HSP70-positive tumor phenotype is associated with a poor clinical outcome, usually correlated with increased resistance to cell death, including resistance to conventional radiotherapy and chemotherapy, as well as aggressive behavior causing early and rapid invasion and metastases. Although it seems that HSP70 expression is linked to T-cell evasion, it has also been shown that membrane-bound HSP70 (mHSP70) serves as a target structure for activated natural killer (NK) cells. Preclinical studies demonstrate that in vitro stimulation of NK cells with an HSP70 peptide plus IL-2 enhances killing of HSP70 membrane-positive tumor cells [46]. Based on this research, a phase I clinical trial was conducted in patients with late-stage colorectal cancer and NSCLC using ex vivo IL-2-stimulated autologous NK cells reinfused [47]. Building on this work, a randomized phase II trial evaluated ex vivo HSP70-stimulated autologous NK cells in patients with NSCLC after radiochemotherapy. The treatment demonstrated a tolerable safety profile and improved progression-free survival compared with standard follow-up, supporting further investigation of HSP70-directed NK-cell immunotherapy [48].

Triptolide is a diterpenoid triepoxide that can be isolated from *T. wilfordii*. In preclinical (in vivo) mouse lung cancer models, combined low-dose aspirin and triptolide reduced spontaneous tumor incidence from 70% to 10% by suppressing NF-κB-driven proliferation in cancer cells through a p53-dependent mechanism, independent of effects on chronic lung inflammation [49]. Additionally, in vivo studies in mouse mesothelioma models demonstrated that triptolide reduced HSP70 levels in a dose-dependent manner [50]. To evaluate its clinical applicability, a phase I trial was conducted to analyze minnelide, a water-soluble disodium salt variant of triptolide, alone and in combination with paclitaxel, a microtubule-stabilizing chemotherapeutic agent, in patients with previously treated gastric cancer. Although single-agent minnelide did not produce objective responses, the addition of paclitaxel improved response outcomes, with one combination regimen demonstrating a disease control rate of 71.4%. These findings suggest that minnelide may enhance paclitaxel sensitivity and support further phase II evaluation of the combination in advanced gastric cancer [51].

Whereas minnelide targets HSP70 expression or activity, other emerging agents aim to directly disrupt HSP70 protein interactions and chaperone function. 2-Phenylethynesulfonamide (PES)-Cl is a modified HSP70 inhibitor derived from 2-phenylethynesulfonamide (PES), distinguished by its added chlorine atom, which enhances its binding, stability, and anticancer effects. Unlike other HSP70 inhibitors, PES-Cl binds to the substrate-binding domain, requires the C-terminal helical lid, and uniquely inhibits anaphase-promoting complex/cyclosome (APC/C) activity. This leads to G2/M arrest, genomic instability, and superior autophagy inhibition, making PES-Cl a promising therapeutic candidate based on preclinical studies with broader mechanisms of action than those of existing HSP70 inhibitors [52]. However, its therapeutic potential remains limited by the absence of clinical trial data, and further investigation is needed to determine its translational applicability.

While early studies suggested that inhibition of HSP70 might produce effects similar to those of HSP90 inhibition, more recent work has demonstrated that selective targeting of HSP70 can yield distinct biological outcomes [53]. In particular, a study by Yaglom et al. utilizing the JG-98 series of HSP70 inhibitors demonstrated that these compounds induce distinct cellular effects that differ significantly from those observed with HSP90 inhibition [54]. Unlike HSP90 inhibitors, which often trigger upregulation of HSP70 and limit therapeutic efficacy, JG-98 uniquely disrupts HSP70 function by targeting its allosteric ATPase domain, leading to dissociation of key cofactors, such as Bag3. This results in widespread dysregulation of cancer-related pathways, including ERK1/2 signaling and proteasome-mediated protein degradation, ultimately promoting apoptosis in tumor cells. Additionally, JG-98 exhibits strong synergy with proteasome inhibitors such as bortezomib, with combination treatment producing significantly greater tumor growth suppression than either agent alone in an in vivo cancer model [54]. Importantly, given that HSP90 inhibition induces compensatory upregulation of HSP70, selective targeting of HSP70 may help overcome this resistance mechanism and enhance therapeutic efficacy. These findings support the notion that selectively targeting HSP70 may overcome the limitations of HSP90 inhibition and provide a more promising strategy for cancer treatment.

### 3.3. HSP110 Inhibitors

Compared with HSP90 and HSP70, therapeutic targeting of HSP110 remains less developed. However, its critical role as a nucleotide exchange factor and growing evidence linking HSP110 to tumor progression and treatment resistance have prompted increasing interest in pharmacologic inhibition.

Several HSP110-targeted therapies remain in the preclinical stage. The first chemical molecular inhibitor of HSP110, named foldamer 33, showed promising results in vitro and in vivo as it prevented cancer cell growth and induced apoptosis in colorectal cancer [55]. Building upon this research, where Foldamer-33 demonstrated efficacy in colorectal cancer models, more recent studies have shown the application of this HSP110 inhibitor, now referred to as iHSP110-33, to hematological malignancies, including primary mediastinal B-cell lymphoma (PMBL) and classical Hodgkin lymphoma (cHL). These studies demonstrated that iHSP110-33 reduced tumor growth and lymphoma cell viability in PMBL and cHL models. Importantly, iHSP110-33 showed synergistic activity when combined with selinexor, an XPO-1-specific nuclear export inhibitor, leading to greater reductions in STAT6 phosphorylation and lymphoma growth in vitro and in vivo than either strategy alone [56]. Further studies have shown that siRNA-mediated silencing of HSP110 in primary effusion lymphoma cells has yielded promising results, in vitro, for the treatment of Kaposi sarcoma herpesvirus-associated lymphoma, which is normally characterized by a poor response to standard chemotherapies. These results can be explained by the role of HSP110 in various cellular processes, including lysosomal permeabilization, DNA repair pathways, and other apoptotic actions [57]. However, despite these promising findings, HSP110-targeted therapies have not yet advanced to clinical trials, and further investigation is required to establish their translational potential.

### 3.4. HSP27 Inhibitors

Alongside HSP90 and HSP70, HSP27 has emerged as a promising anticancer target due to its role as a master regulator of multiple oncoproteins and its contribution to tumorigenic and pro-survival pathways [58]. However, therapeutic development targeting HSP27 has remained comparatively limited. Representative strategies to disrupt HSP27-mediated survival mechanisms are discussed below.

Among clinically evaluated approaches, the most extensively studied therapy is OGX-427 (apatorsen), an antisense oligonucleotide that targets HSP27. In a phase I dose escalation study of patients with castration-resistant prostate cancer and other advanced cancers, apatorsen demonstrated acceptable tolerability and evidence of biological activity as a single agent [59]. Building on this rationale, Borealis-1 evaluated apatorsen in combination with chemotherapy as a first-line treatment for advanced urothelial cancer. In this combination strategy, apatorsen was intended to suppress HSP27-mediated stress survival signaling, while chemotherapy targeted rapidly dividing tumor cells. However, despite acceptable tolerability, Borealis-1 did not demonstrate significant improvement in overall outcomes [60]. Borealis-2 subsequently evaluated apatorsen plus docetaxel, a microtubule-stabilizing taxane, versus docetaxel in platinum-resistant metastatic urothelial carcinoma. This study showed a modest overall survival benefit with the combination, although progress-free survival and objective response rates were similar, highlighting mixed clinical benefit despite the biological rationale [61].

Another example is Compound I, a small-molecule HSP27 inhibitor that drives proteasomal degradation of both mutant and wild-type androgen receptors (AR) in glioblastoma (GBM) cells, selectively inhibiting AR-overexpressing GBM growth at low nanomolar concentrations [62]. In preclinical (in vivo) studies, it significantly reduces tumor burden at 20 mg/kg without observable toxicity up to 80 mg/kg, highlighting HSP27 targeting as a promising therapeutic strategy. However, this compound remains in the preclinical stage, and further studies are required to determine its clinical applicability.

### 3.5. HSP60 Inhibitors

Therapeutic targeting of HSP60 remains largely preclinical, though growing recognition of its role in mitochondrial function and tumor biology has prompted interest in HSP60-directed strategies. Representative natural inhibitors with emerging anticancer potential are discussed below.

For example, preclinical studies (in vitro) have shown that curcumin, a natural anti-inflammatory compound, induces apoptosis in neuroblastoma cells by modulating HSP60 expression, localization, and post-translational modifications [63]. At higher doses, curcumin decreased HSP60 protein levels, enhanced its folding activity, promoted secretion, and disrupted the HSP60/HSP10 machinery, suggesting a protective role against cancer progression. However, the clinical translation of curcumin is limited by its poor oral bioavailability, which restricts its therapeutic applicability despite promising preclinical findings [64].

Furthermore, myrtucommulone A (MC) has been shown to directly target the mitochondrial chaperonin HSP60, disrupting its protein-folding function and inducing mitochondrial dysfunction in cancer cells. MC binding to HSP60 leads to impaired refolding activity and protein aggregation under stress conditions [65]. These findings further highlight HSP60 as a potential mediator of MC-induced apoptosis and a possible therapeutic agent in cancer. However, these findings again remain limited to the preclinical setting, and further studies are necessary to evaluate their clinical application.

### 3.6. HSP Vaccines

Another way heat shock proteins have been used in cancer treatment is through HSP-based vaccines. HSP vaccines are a promising form of tailored immunotherapy that leverages the innate chaperoning function of HSPs to present tumor-derived peptides to the immune system. With HSP/peptide complexes, these vaccines effectively create a greater antitumor immune response without the need to identify an individual tumor antigen. In this section, HSP-based vaccines are discussed with emphasis on their mechanism of action, clinical development stage, and observed therapeutic outcomes.

Currently, the most promising of these vaccines is Vitespen, an HSPPC-96-based vaccine. It has been shown to exhibit immunogenicity in various malignancies with phase III trials including kidney cancer and melanoma [66]. While overall survival between groups was not significantly different in the melanoma trial cohort, exploratory analyses indicated that patients with less advanced disease (M1a/M1b) who received ≥10 doses of Vitespen showed improved survival, supporting a delayed immunologic response and identifying a subgroup that may benefit from further study [67].

Similarly, viagenpumatucel-L (HS-110) is another heat shock protein-based allogeneic cell-based cancer vaccine that was initially derived for use in the treatment of advanced non-small cell lung cancer. It uses a target-delivery system that delivers a modified, secretable form of the heat shock protein gp96, which presents tumor-associated antigens to the patient’s antigen-presenting cells via the MHC-1 cross-presentation pathway [68]. This pathway promotes strong activation of CD8+ cytotoxic T-cells that effectively address tumor heterogeneity and overcome MHC restrictions, helping distinguish the vaccine from earlier peptide- and MHC-focused vaccines. A phase II study investigating the safety of HS-110 in combination with nivolumab, a PD-1 immune checkpoint inhibitor that enhances T-cell-mediated antitumor activity, was conducted in patients with advanced NSCLC, including both checkpoint inhibitor (CPI)-naive and CPI-experienced cohorts. Interim results showed that the vaccine was well tolerated and suggested clinical activity in selected patient subgroups [69]. Taken together, these two heat shock protein-based vaccines demonstrate encouraging clinical activity and safety, highlighting the potential of HSP-targeted immunotherapies in cancer treatment. However, these findings remain preliminary, and further studies are required to establish their clinical efficacy.

## 4. Discussion

Cancer cells are exposed to significant external stressors in the tumor microenvironment, which, in turn, upregulate the expression of various HSPs (Table 2). Thus, heat shock proteins are frequently overexpressed in various tumors and have been implicated in tumorigenesis, invasion, metastasis, and immune recognition [70]. For instance, HSP90 is secreted by tumor cells and activates metalloproteinase-2 (MMP-2), which works to digest the extracellular matrix. Through this mechanism, HSP90 is highly implicated in enhancing cancer cell invasiveness [15].

Based on these favorable preclinical data, the HSP70 and HSP90 protein families have been studied in clinical trials as targets for anti-tumorigenesis therapeutics [71]. Both families are encoded by the HSPA gene family. HSP70 has thirteen members, and HSP90 has five members [71]. Both families have been implicated in the tumorigenesis of multiple subtypes of malignancies, including pancreaticobiliary, gastric, colorectal, gynecological, breast, and prostate cancer. HSPs have also been implicated in resistance to certain therapies. For example, HSPJ2, a member of the HSP70 family, has been linked to resistance to platinum agents and proteasome inhibitors [71].

In breast cancer, high levels of expression of certain heat shock proteins, such as HSP90AA1, correlated with higher risk breast cancer subtypes. Higher subtypes were defined as larger tumors (high T stage), higher grade, lymph node involvement, hormone receptor-negative, and HER2/neu-Positive [72]. Similarly, immunohistochemistry scoring of HSP90AA1 in invasive breast cancer revealed that increased protein expression correlated with higher grade, evidence of lymphovascular invasion, hormone receptor-negative tumors, HER2neu-positive tumors, luminal B subtype, triple-negative breast cancer, PIK3CA-mutated tumors, and pTP53 tumors [73]. These findings suggest that dysregulation of heat shock proteins contributes to tumor aggressiveness and adverse clinical outcomes.

Despite strong preclinical rationale, the clinical translation of HSP90-targeted therapies has been limited. Most developed HSP90 inhibitors target the N-terminal nucleotide-binding domain of the protein, which is responsible for ATP hydrolysis. Inhibition of ATP hydrolysis interferes with protein folding, and non-conformed HSP90 proteins are then degraded via proteasomes. This degradation process in turn activates the heat shock response, leading to increased transcription of all HSPs, including HSP90 [74]. This compensatory upregulation of HSPs represents a key mechanism of resistance that limits the therapeutic efficacy of HSP90 inhibition.

Inhibition of the C-terminal nucleotide-binding domain, albeit a less common target in therapeutic development, induces HSP90 degradation without triggering the heat shock response pathway, highlighting an alternative strategy to mitigate this limitation [74]. More broadly, these findings suggest that targeting individual HSPs in isolation may be insufficient. Instead, combination or multi-targeted approaches, particularly those simultaneously targeting HSP90 and HSP70, may be required to overcome compensatory survival pathways and achieve more durable therapeutic responses.

## 5. Conclusions

Heat shock proteins may serve as targets for single-agent, combination, or vaccine therapies. The role of HSP70 in promoting cell survival and its overexpression in various cancers underscore its potential as a therapeutic target. Preclinical and early-phase clinical studies have demonstrated promising outcomes, including downregulation of HSP70, triggering apoptosis, and enhancing NK cell-mediated cytotoxicity against HSP70-expressing tumor cells. Furthermore, compounds such as triptolide, along with their derivatives, have shown encouraging safety profiles and antitumor activity in early trials. These findings suggest that HSP70 inhibitors hold significant promise for future clinical applications, particularly in cancers where HSP70 is overexpressed and contributes to tumor progression and therapy resistance.

Furthermore, promising preclinical data suggest that the combination of HSP90 and HSP70 inhibitors provides a more effective tumor cytotoxic effect without compensatory activation of the heat shock response, supporting a multi-targeted therapeutic approach. In contrast, both HSP27 and HSP110 inhibitors have limited to no clinical studies and remain largely in the preclinical stage. Given their functional interplay with HSP70, these proteins may represent additional targets within the heat shock protein network, although further studies are required to clarify their therapeutic role.

In addition to small-molecule inhibitors, heat shock protein-based vaccines have emerged as a complementary strategy, leveraging the chaperone function of HSPs to enhance tumor antigen presentation and stimulate antitumor immune responses. While early clinical studies have demonstrated immunogenicity, further investigation is needed to determine the safety and efficacy of these products. Collectively, these findings support the continued exploration of heat shock protein-targeted therapies, particularly through combination and multi-modal approaches, to overcome resistance mechanisms and improve clinical outcomes.

## 6. Future Directions

Future therapeutic strategies targeting heat shock proteins should aim to address the limitations observed with current approaches. HSP90 inhibitors currently in development target multiple HSP90 isoforms; however, isoform-specific inhibitors may lead to more targeted and less toxic treatments, as different isoforms may have distinct roles in specific cancer types or stages.

While HSP90 has been a primary focus, other HSPs such as HSP27, HSP70, and small HSPs (sHSPs) are also gaining attention as potential therapeutic targets. Given the compensatory upregulation of HSP70 following HSP90 inhibition, future approaches may benefit from combination or multi-targeted strategies that simultaneously disrupt multiple components of the heat shock protein network.

Further areas of investigation include focusing on (1) developing isoform-specific HSP90 inhibitors, (2) leveraging HSPs as adjuvants in immunotherapy, (3) understanding HSP regulation through post-translational modifications, (4) elucidating how HSPs contribute to chemoresistance by stabilizing or upregulating drug efflux pumps, and (5) identifying synergistic effects of HSP inhibitors combined with other anticancer therapies, such as chemotherapy and radiotherapy.

## Figures and Tables

**Table 1:** Major heat shock protein families: localization and function.

HSP Family	Primary Location	Function	References
HSP27	Cytoplasm	ATP-independent chaperone involved in cell motility, cytoskeletal stabilization, and tumor invasion	[5,6]
HSP60	Mitochondria	Mitochondrial chaperone that regulates oxidative stress and supports mitochondrial protein folding and survival	[7]
HSP70	Cytoplasm	ATP-dependent chaperone that inhibits apoptosis (e.g., BAX, AIF), regulates stress signaling, and promotes tumor cell survival	[8]
HSP90	Cytoplasm	ATP-dependent chaperone that stabilizes oncogenic client proteins and regulates key signaling pathways involved in cell growth and survival	[9,10,11]
HSP 110	Cytoplasm	Nucleotide exchange factor for HSP70 that enhances protein disaggregation and stress recovery	[12,13]

BAX = Bcl-2-associated X apoptosis regular, AIF = apoptosis-inducing factor.

**Table 2:** Heat shock protein-targeted therapies in cancer.

Inhibitor	Stage of Evaluation	Cancer	N	Outcomes/Limitations
HSP27				
Apatorsen	Phase I	Solid tumors	42	Well tolerated; mostly grade 1–2 AEs; no MTD reached; modest biological activity with stable disease and PSA/CTC reductions
Apatorsen + gemcitabine + cisplatin	Phase II (Borealis-1)	Urothelial carcinoma	183	No OS benefit overall; manageable toxicity; possible benefit in poor-prognosis subgroup
Apatorsen + docetaxel	Phase II (Borealis-2)	Urothelial carcinoma	200	Modest OS improvement; no PFS/ORR benefit; increased infection risk
Compound I [62]	Preclinical	Glioblastoma models	—	Inhibits androgen receptor signaling; reduces tumor growth in preclinical models
HSP60				
Curcumin	Preclinical	Neuroblastoma models	—	Modulates HSP60 expression and function; disrupts HSP60/HSP10 complex; induces apoptosis; limited by poor bioavailability
Myrtucommulone A	Preclinical	Cancer cell models	—	Directly targets HSP60; inhibits chaperone function; induces mitochondrial dysfunction and apoptosis
HSP70				
HSP70-stimulated NK cells	Phase I	Colorectal cancer, NSCLC	12	Well tolerated; enhanced NK cytotoxic activity; limited clinical response (stable disease)
HSP70-stimulated NK cells	Phase II	NSCLC	16	Well tolerated; improved PFS vs. control (not statistically significant); increased NK activation; small sample size
Minnelide	Phase I	Gastric cancer	36	Well tolerated; no objective responses with monotherapy; improved response with paclitaxel; overlapping toxicities; small sample size
Triptolide	Preclinical	Lung cancer, mesothelioma models	—	Reduces HSP70 levels; inhibits NF-κB signaling; induces apoptosis; suppresses tumor growth
PES-Cl (pifithrin-μ)	Preclinical	Cancer cell models	—	Disrupts HSP70 function; induces G2/M arrest and autophagy inhibition; antitumor activity in vivo
JG-98	Preclinical	Cancer cell models	—	Disrupts HSP70-Bag3 interaction; alters oncogenic signaling; induces apoptosis; synergizes with targeted therapies
HSP90				
17-AAG + Trastuzumab	Phase II	HER2+ breast cancer	31	Objective responses observed; clinical benefit rate 59%; mostly grade 1 toxicities
17-AAG + Sorafenib	Phase I	Kidney cancer, melanoma	27	Clinical and pharmacodynamic activity observed; dose-limiting transaminitis and hand-foot syndrome
Ganetespib + docetaxel	Phase III	NSCLC	677	No improvement in OS or PFS vs. docetaxel; trial stopped early for futility; neutropenia common
Onalespib + AT7519	Phase I	Solid tumors	28	Tolerable; pharmacodynamic target engagement; preliminary clinical activity with rare partial responses
Onalespib + erlotinib	Phase I/II	NSCLC	11	Limited clinical activity; no objective response; overlapping toxicities (diarrhea); trial closed early
Pimitespib	Phase I	Solid tumors	61	Acceptable safety profile; preliminary antitumor activity with partial responses observed
Pimitespib	Phase III	GIST	86	Improved PFS and overall survival; acceptable safety profile
NCT-58	Preclinical	Breast cancer models	—	Reduces tumor growth and stem-like markers; may overcome trastuzumab resistance
HSP110				
Foldamer 33 (iHSP110-33)	Preclinical	Colorectal cancer, lymphoma models	—	Induces apoptosis and reduces tumor growth; synergizes with selinexor
HSP Vaccines				
Vitespen (HSPPC-96)	Phase III	Melanoma	322	No overall survival benefit; minimal toxicity; possible benefit in less advanced disease subgroups
Viagenpumatucel-L	Phase II (Interim)	NSCLC	115	Well tolerated; preliminary clinical activity in selected subgroups

MTD, maximum tolerated dose; PSA, prostate-specific antigen; CTC, circulating tumor cells; OS, overall survival; PFS, progression-free survival; ORR, overall response rate; NSCLC, non-small cell lung cancer; PES-Cl, 2-Phenylethynesulfonamide-chlorine; “—” indicates preclinical studies for which no human sample size was applicable.

## Data Availability

No new data were created or analyzed in this study. Data sharing is not applicable to this article.

## Abbreviations

The following abbreviations are used in this manuscript:

AR	Androgen receptor
cHL	Classical Hodgkin lymphoma
CPI	Checkpoint inhibitor
GBM	Glioblastoma
GIST	Gastrointestinal stromal tumors
HS-110	Viagenpumatucel-L
HSP	Heat shock protein
HSP40	Co-chaperones in the J protein family
HSR	Heat shock response
kDA	KiloDalton
MC	Myrtucommulone
MMP-2	Metalloproteinase-2
NK	Natural killer
NSCLC	Non-small cell lung carcinoma
PDAC	Pancreatic ductal adenocarcinoma
PES	2-Phenylethynesulfonamide
PES-Cl	2-Phenylethynesulfonamide-Cl
PMBL	Primary mediastinal B-cell lymphoma
sHEPs	Small heat shock proteins
STA-9090	Ganetespib
TAS-116	Pimitespib
